# Delayed endoscopic necrosectomy improves hospital length of stay and reduces endoscopic interventions in patients with symptomatic walled‐off necrosis

**DOI:** 10.1002/deo2.162

**Published:** 2022-09-08

**Authors:** Rishi Pawa, Robert Dorrell, Clancy Clark, Greg Russell, John Gilliam, Swati Pawa

**Affiliations:** ^1^ Department of Medicine, Division of Gastroenterology Wake Forest University School of Medicine Winston‐Salem USA; ^2^ Department of Medicine Wake Forest University School of Medicine Winston‐Salem USA; ^3^ Department of General Surgery Wake Forest University School of Medicine Winston‐Salem USA; ^4^ Department of Biostatistics and Data Science Wake Forest University School of Medicine Winston‐Salem USA

**Keywords:** acute necrotizing, endoscopic, endoscopic ultrasonography, gastrointestinal surgery, metallic stents, pancreatitis, self‐expandable

## Abstract

**Objectives:**

Advancements in the endoscopic management of walled‐off necrosis using lumen apposing metal stents have improved outcomes over its surgical and percutaneous alternatives. The ideal procedural technique and timing of direct endoscopic necrosectomy (DEN) have yet to be clarified.

**Methods:**

From November 2015 to June 2021, a retrospective comparative cohort analysis was performed comparing clinical outcomes for patients undergoing immediate DEN (iDEN) versus delayed DEN (dDEN). Subgroups were identified based on the quantification of necrosis. Wilcoxon two‐sample tests were used to compare continuous variables and Fisher's exact test was used to compare categorical variables.

**Results:**

A total of 80 patients underwent DEN for management of walled‐off necrosis (iDEN = 43, dDEN = 37). Technical success was achieved in all patients. Clinical success was seen in 39 (91%) patients in the iDEN group and 34 (92%) in the dDEN group. Amongst iDEN patients, the mean number of necrosectomies was 2.5 (standard deviation [SD] 1.4) in comparison to 1.5 (SD 1.0) for dDEN (*p*‐value = 0.0011). The median index hospital length of stay was longer with iDEN than dDEN (7.5 days vs. 3.0 days respectively, *p*‐value = 0.010). Subgroup analysis was performed based on the percentage of necrosis (<25% vs. >25% necrosis). iDEN was associated with more necrosectomies than dDEN regardless of the percentage of necrosis (*p* = 0.017 and 0.0067, respectively).

**Conclusion:**

Patients undergoing dDEN had a shorter index hospital stay and fewer necrosectomies than iDEN. The large diameter of lumen apposing metal stents permits adequate drainage allowing a less aggressive approach thereby improving clinical outcomes and avoiding unnecessary interventions.

## INTRODUCTION

Walled‐off necrosis (WON) is a late sequela of acute necrotizing pancreatitis characterized by the development of a mature encapsulated necrotic collection with a well‐defined wall.^1^ These collections require drainage when patients develop symptoms including, but not limited to, abdominal pain, fever, nausea, vomiting from gastric outlet obstruction, and jaundice from biliary obstruction.^2^ While the mortality associated with WON can reach 10% in sterile collections, fatality increases to 30% with the presence of infection.^3^ Various approaches have been utilized to drain these collections including endoscopic, percutaneous, and surgical drainage.^4^

Direct endoscopic necrosectomy (DEN) was first described by Seifert et al. in 2000 with a case series of three patients who underwent endoscopic management of WON.^5^ The PANTER trial in 2010 resulted in a paradigm shift in the management of infected WON with a significant decrease in major complications and healthcare utilization in the step‐up group versus open surgical necrosectomy.^6^ Over the last decade, numerous studies have favored endoscopic necrosectomy over percutaneous and surgical interventions due to its high efficacy, shorter hospital length of stay, lower reintervention rates, and improved clinical outcomes.^7^, ^8^ Lumen apposing metal stents (LAMS) have further improved management of WON given their ease of deployment and shorter procedure time coupled with improved drainage due to their large diameter in comparison to plastic stents.^9^ In addition, once the LAMS is deployed it can be used as a port, permitting passage of an endoscope inside the cyst cavity and allowing direct endoscopic visualization and debridement of necrosis using endoscopic tools such as rat tooth forceps, snares, baskets, and a morcellating device.^10^

While endoscopic necrosectomy has been placed at the forefront of WON management, the optimal endoscopic approach has yet to be clarified.^11^ Limited studies are currently available on DEN as existing data has relied on multicenter trials in which significant heterogeneity is present in providers’ procedural techniques and skills.^12^ In addition, the timing of DEN is unclear with some endoscopists performing DEN at the time of LAMS placement and others delaying it. We present our single‐center experience of endoscopic management of WON and summarize the optimal approach.

## METHODS

### Patients

**FIGURE 1:**
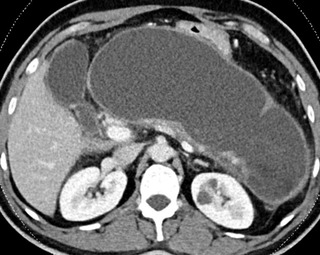
Computed tomography of large walled‐off necrosis prior to endoscopic intervention

From November 2015 to June 2021, an IRB‐approved retrospective comparative cohort analysis was performed for patients with symptomatic WON undergoing endoscopic necrosectomy using LAMS. WON was defined as a mature encapsulated collection with necrotic material greater than 4 weeks old after the onset of necrotizing pancreatitis. The diagnosis of WON and quantification of necrosis were made using either computed tomography or magnetic resonance imaging by an experienced abdominal radiologist (Figure 1). This diagnosis was subsequently confirmed on endoscopic ultrasound. Patients underwent DEN if they developed symptoms related to the collection. These symptoms included but were not limited to abdominal pain, nausea, and vomiting related to gastric outlet obstruction, jaundice secondary to biliary obstruction, or fever due to infection.

Inclusion criteria included patients who underwent endoscopic necrosectomy for symptomatic WON (with greater than 10% necrosis) using LAMS and follow‐up duration of at least 6 months from the index procedure. Patients less than 18 years of age, presence of pancreatic malignancy, postoperative fluid collections, WON with less than 10% necrosis, an international normalized ratio greater than 1.5, and platelet count less than 50,000 were excluded from the study. Patient demographics, collection characteristics, procedural technique, and clinical outcomes were recorded and reviewed. Subgroups were identified based on the percentage of necrosis (<25% vs. >25%) and timing of endoscopic necrosectomy. Patients either underwent necrosectomy at the time of LAMS placement (initial direct endoscopic necrosectomy, iDEN) or delayed debridement on subsequent endoscopy at least 1 week after LAMS placement (delayed DEN, dDEN).

### Procedural technique

**FIGURE 2:**
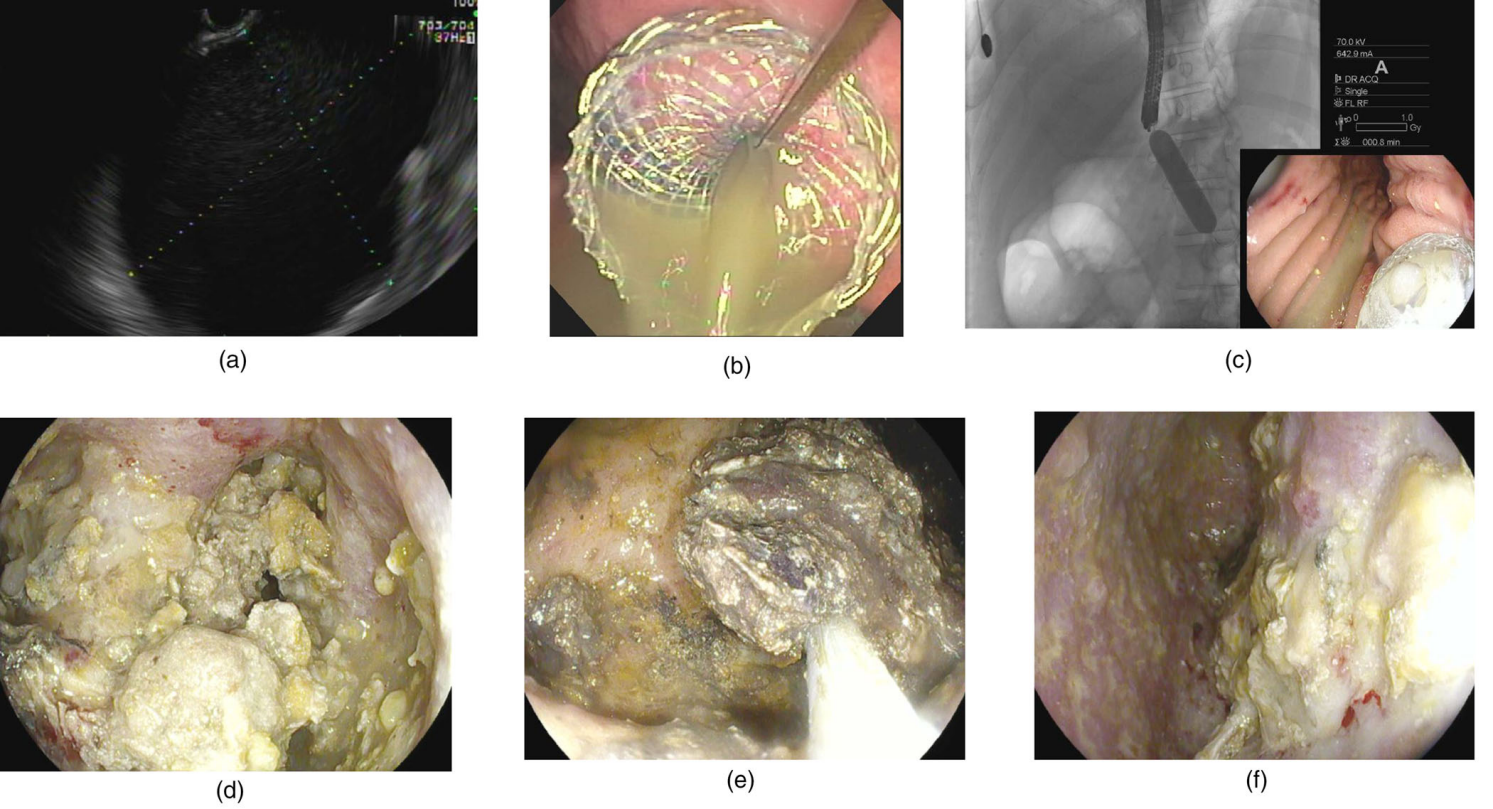
Endoscopic management of walled‐off necrosis. (a) Endoscopic ultrasound imaging of walled‐off necrosis. (b) Endoscopic visualization of lumen apposing metal stent after deployment in the cyst cavity with drainage of purulent fluid. (c) Balloon dilatation of lumen apposing metal stent after deployment under endoscopic and fluoroscopic guidance. (d) Endoscopic visualization of necrosis in the cyst cavity prior to direct endoscopic necrosectomy. (e) Direct endoscopic necrosectomy using a snare. (f) Clean cyst cavity after direct endoscopic necrosectomy

All patients consented to the procedure and agreed to participate in the study. General anesthesia was performed on all patients. Intravenous antibiotics were given at the initiation of the procedure. The collection was visualized using a therapeutic linear echoendoscope (Olympus, Tokyo, Japan) from the stomach or duodenum, depending on the location of the collection. Endoscopic ultrasound imaging with a doppler was used to determine the safest deployment tract for LAMS. A 15 × 10 mm LAMS (Boston Scientific, Marlborough, MA) was used in all patients. Following deployment, the stent was dilated to its diameter using a through‐the‐scope dilating balloon. All patients underwent hydrogen peroxide lavage with 100 milliliters at 0.3% concentration at the time of initial LAMS placement and subsequent necrosectomies. No patients underwent double pigtail plastic stent placement through the LAMS. Patients with systemic inflammatory response syndrome (SIRS) within 1 week prior to endoscopic drainage underwent delayed necrosectomy (dDEN) at least 1 week after initial stent placement. If SIRS was absent in the week prior to endoscopic drainage, necrosectomy was performed at the time of initial LAMS placement (iDEN). The reason for delayed necrosectomy in patients with SIRS within a week was to allow the inflammatory response to dampen after performing initial drainage using LAMS. DEN was performed using snares and rat tooth forceps (Figure 2a–f). All patients undergoing endoscopic drainage were kept off proton pump inhibitors from the time of LAMS placement to the time of removal.

Follow‐up imaging was performed at 1 week, 3–4 weeks, and 6 weeks following initial stent placement and 1 week after every necrosectomy. Patients showing a lack of improvement in symptoms with persistent necrotic material in the cyst cavity as seen on imaging underwent repeat necrosectomy. All patients underwent follow‐up imaging at 6 months to detect recurrence. LAMS was removed once the patient achieved adequate debridement and drainage with a resolution of symptoms. For patients showing clinical deterioration despite endoscopic necrosectomy and initiation of antibiotics, a step‐up approach was utilized including percutaneous drainage, video‐assisted retroperitoneal debridement, or surgical open necrosectomy as the last resort.

### Clinical outcomes

Technical success was defined as complete clearance of necrotic material from the cyst cavity via an endoscopic approach without escalation to percutaneous drainage or surgical necrosectomy. Clinical success was defined as resolution of symptoms and collection size less than or equal to 2 cm on cross‐sectional abdominal imaging at the time of LAMS removal. Recurrence was defined as reaccumulation of the fluid collection at the same location (greater than 2 cm in size) on follow‐up imaging. Follow‐up duration was determined from the time of LAMS placement to the last clinic visit with a gastroenterologist. Adverse events were characterized as immediate (within 48 h of the procedure) or late (more than 48 h post‐procedure) and graded according to the American Society of Gastrointestinal Endoscopy Lexicon criteria.

The primary outcome was comparing clinical success in the two groups (iDEN vs. dDEN). Secondary outcomes included technical success, number of endoscopic interventions required to achieve WON resolution, index hospital length of stay (initial hospital length of stay following LAMS placement), adverse events, mortality, and recurrences.

### Statistical analysis

Descriptive statistics were calculated for continuous (mean, standard deviation (SD), median, and interquartile range [IQR]) and categorical variables (frequency and proportion). Group comparisons were calculated using independent *t*‐tests and Wilcoxon two‐sample tests for continuous variables and Fisher's Exact Tests for categorical data. The median follow‐up and its corresponding 95% confidence interval were calculated using the Kaplan‐Meier estimator. Statistical significance was assumed if the *p*‐value was less than 0.05. SAS (version 9.4; Cary, NC, USA) was used for all analyses.

## RESULTS

From November 2015 to June 2021, 115 patients diagnosed with symptomatic pancreatic fluid collections underwent endoscopic management with LAMS. Of these patients, 80 were found to have WON. Forty‐three patients had DEN performed at the time of LAMS placement (iDEN) and 37 patients had DEN deferred to later endoscopy (dDEN).

The average age of patients was 54.7 years (SD 14.8), and 31 were female (38.8%). The etiologies of pancreatitis included: gallstone (*n* = 33, 41.3%), alcohol (*n* = 25, 31.0%), idiopathic (*n* = 16, 20.0%), hypertriglyceridemia (*n* = 4, 5.0%), and medication‐induced (*n* = 2, 2.5%). Hemorrhage was present in 13 collections (16.3%) and infection was present in 34 collections (42.5%). Of the 43 patients in the iDEN group, 18 had 11%–25% necrosis, 13 had 26%–50% necrosis, and 12 had greater than 50% necrosis. Of the 37 patients in the dDEN group, 21 patients had necrosis between 11%–25%, eight had necrosis between 26%–50%, and eight had greater than 50% necrosis. Patient demographics, collection characteristics, and outcomes are summarized in Tables 1, 2, 3.

**TABLE 1:** Demographics and collection characteristics of patients who underwent endoscopic management of symptomatic walled‐off necrosis

**Patients with WON (*n* = 80)**	**Immediate** **DEN (*n* = 43**)	**Delayed DEN (*n* = 37)**	***p*‐value**
Mean age (SD)	52.2 (15.4)	57.5 (13.8)	0.11
Female gender	19 (44%)	12 (32%)	0.36
Etiology of pancreatitis			0.30
Gallstone	21 (49%)	12 (32%)	
Alcoholic	10 (23%)	15 (41%)	
Idiopathic	8 (18%)	8 (22%)	
Hypertriglyceridemia	2 (5%)	2 (5%)	
Medication‐induced	2 (5%)	0 (0%)	
Mean BMI (kg/m^2^) (SD)	29.3 (7.2)	28.2 (6.7)	0.48
Mean area of collection (cm^2^) (SD)	128.4 (107.0)	114.7 (79.4)	0.52
Hemorrhage	6 (14%)	7 (19%)	0.56
Infection, *n* (%)	20 (47%)	14 (38%)	0.50
Paracolic gutter extension, *n* (%)	12 (28%)	9 (24%)	0.80
Endoscopic approach			0.17
Transgastric	41 (95%)	31 (84%)	
Transduodenal	2 (5%)	5 (14%)	
Transesophageal	0 (0%)	1 (3%)	
Percentage necrosis			0.26
<25	18	21	
>25	25	16	

Abbreviations: BMI, body mass index; dDEN, delayed direct endoscopic necrosectomy; iDEN, immediate direct endoscopic necrosectomy; SD, standard deviation; WON, walled‐off necrosis.

**TABLE 2:** Clinical outcomes of patients who underwent endoscopic management of symptomatic walled‐off necrosis

**Patients with WON (*n* = 80)**	**iDEN (*n* = 43)**	**dDEN (*n* = 37)**	***p*‐value**
Length of follow‐up, days (median, IQR)	488 (350, 580)	609 (456, 1018)	0.50
Length of hospitalization, days (median, IQR)	7.5 (2, 21)	3 (1, 7)	0.01
Need for concomitant percutaneous IR drainage, *n* (%)	6 (14%)	6 (16%)	>0.99
Number of percutaneous procedures until the last follow‐up, mean (SD)	1.6 (5.9)	1.3 (3.1)	0.81
Technical success of necrosectomy, *n* (%)	40 (93%)	34 (92%)	>0.99
Mean procedure time (min)	54.0 (23.7)	38.7 (17.3)	0.0017
Mean duration of LAMS placement (days)	40.0 (24.7)	44.3 (24.0)	0.45
Mean number of necrosectomies (mean, SD)	2.5 (1.4)	1.5 (1.0)	0.0011
Clinical success, *n* (%)	39 (91%)	34 (92%)	>0.99
Recurrence of PFC at 6 months, *n* (%)	0/36 (0%)	3/33 (9%)	0.10
Adverse events, *n* (%)	5 (12%)	5 (14%)	>0.99
Early (<48 h)			
SIRS	2	0	
Late			
Stent occlusion	2	3	
Stent migration	0	1	
Bleeding	1	1	
Total mortality at 6 months, *n* (%)	7 (16%)	4 (11%)	0.53

Abbreviations: dDEN, delayed direct endoscopic necrosectomy; iDEN, immediate direct endoscopic necrosectomy; IR, interventional radiology; LAMS, lumen apposing metal stents; IQR, interquartile range; PFC, pancreatic fluid collection; SIRS, systemic inflammatory response syndrome; SD, standard deviation; WON, walled‐off necrosis.

**TABLE 3:** Comparative metrics of patients in the immediate direct endoscopic necrosectomy and delayed DEN groups

**Patients with WON (*n* = 80)**	**iDEN (*n* = 43)**	**dDEN (*n* = 37)**	***p*‐value**
Median Charlson Comorbidity Index (IQR)	2 (1,5)	3 (1.5, 5)	0.16
Median ASA (IQR)	3 (3, 3)	3 (3, 3)	>0.99
Revised Atlanta classification			0.64
Moderately severe, *n* (%)	27 (63%)	26 (70%)	
Severe, *n* (%)	16 (37%)	11 (30%)	
Systemic inflammatory response syndrome, *n* (%)	41 (95%)	37 (100%)	0.50
Single organ failure prior to drainage, *n* (%)	13 (30%)	13 (35%)	0.81
Multi‐organ failure prior to drainage, *n* (%)	7 (16%)	5 (14%)	0.76
ICU admission prior to intervention, *n* (%)	15 (35%)	11 (30%)	0.64
Single organ failure after drainage, *n* (%)	7 (16%)	5 (14%)	0.20
New onset	3	0	
Persistent	4	5	
Multi‐organ failure after drainage, *n* (%)	3 (7%)	2 (5%)	>0.99
New onset	0	0	
Persistent	3	2	
ICU admission after the intervention, *n* (%)	5 (12%)	3 (8%)	>0.99
New onset	1	0	
Persistent	4	3	

Abbreviations: ASA, American Society of Anesthesiologists; dDEN, delayed direct endoscopic necrosectomy; ICU, intensive care unit; iDEN, immediate direct endoscopic necrosectomy; IQR, interquartile range; WON, walled‐off necrosis.

All patients in the iDEN and dDEN groups had successful LAMS placement for DEN. Technical success was achieved in 40/43 (93%) patients in the iDEN group and 34/37 (92%) in the dDEN group (*p* ≥ 0.99). Clinical success was seen in 39/43 (91%) patients in the iDEN group and 34/37 (92%) in the dDEN group (*p* ≥ 0.99). Of the four clinical failures in the iDEN group, two patients died within 2 weeks of the procedure due to worsening septic shock despite additional percutaneous drainage. In the third patient, management was stepped up to surgical necrosectomy due to ongoing sepsis; however, the patient passed away 116 days later from multiorgan failure. The fourth patient developed a pancreaticopleural fistula and parapneumonic effusion and died 119 days after LAMS placement. Of the three clinical failures in the dDEN group, two patients underwent step‐up surgical necrosectomy due to a lack of improvement after endoscopic drainage and DEN. In one patient, percutaneous drainage was performed prior to surgical necrosectomy. The second patient was taken directly to surgery due to a lack of a safe window for percutaneous drain placement. None of these patients had a separate collection. Both patients fully recovered and were sent home after surgery. The third patient was discharged to hospice after LAMS placement and delayed necrosectomy (1 week after LAMS placement). Given the lack of clinical improvement, the patient decided to forego any further intervention and elected hospice care. The stent was never removed, and the patient passed away 50 days later.

**FIGURE 3:**
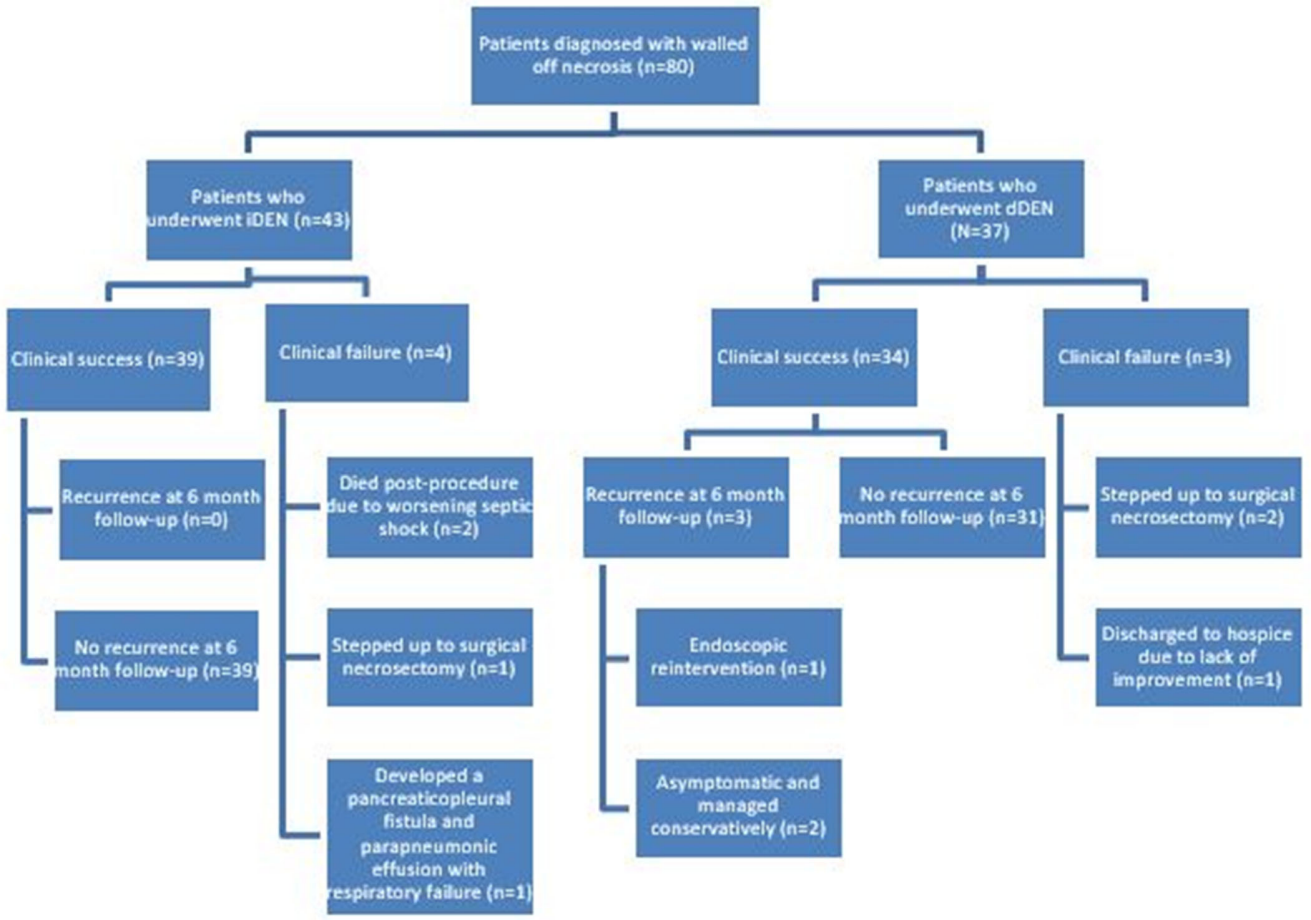
Flow chart of patients who underwent endoscopic management of walled‐off necrosis

In the iDEN group, 10 patients had one necrosectomy session, 18 patients had two sessions, five patients had three sessions, and 10 patients had four or more sessions. In the dDEN group, 27 patients had one necrosectomy session, four patients had two sessions, three patients had three sessions, and three patients had four or more sessions. The median time to initial necrosectomy after LAMS placement in the delayed group was 7 days (IQR 7, 8). The mean number of necrosectomies for patients who underwent iDEN was 2.5 (SD 1.4) in comparison to 1.5 (SD 1.0) for dDEN (*p*‐value = 0.0011). The median index hospital length of stay was longer in the iDEN cohort versus the dDEN cohort (7.5 days vs. 3.0 days respectively, *p*‐value = 0.010; Figure 3).

The patients were further subdivided into two subgroups (<25% vs. >25% necrosis) based on the quantification of necrosis seen on initial imaging (Table 4). In patients with <25% necrosis, iDEN was associated with more necrosectomies than dDEN (median 2 vs. 1, *p* = 0.017). The length of hospitalization was not significantly different between the two groups (*p* = 0.11). In patients with >25% necrosis, iDEN was similarly associated with more necrosectomies than dDEN (median 2 vs. 1, *p* = 0.0067). In this cohort, patients undergoing iDEN had a longer index hospital stay than dDEN though the results were not statistically significant (median index length of stay 14 vs. 3, *p* = 0.066).

**TABLE 4:** Subgroup analysis based on the percentage of necrosis in the cyst cavity

**Subgroups analysis on quantification of necrosis**			
**≤25% necrosis (*n* = 39)**	**iDEN (*n* = 18)**	**dDEN (*n* = 21)**	***p*‐value**
Median index length of stay (days)	4	2	0.11
Median number of necrosectomies	2	1	0.017

**≥25% necrosis (*n* = 41)**	**iDEN (*n* = 25)**	**dDEN (*n* = 16)**	***p*‐value**
Median index length of stay (days)	14	3	0.066
Median number of necrosectomies	2	1	0.0067

Abbreviations: dDEN, delayed direct endoscopic necrosectomy; iDEN, immediate direct endoscopic necrosectomy.

There were ten procedure‐related adverse events in the study cohort. In the iDEN group, two patients experienced systemic inflammatory response syndrome within 12 h of the procedure, requiring overnight ICU admission. Late adverse events in the iDEN group included stent occlusion (*n* = 2) and self‐limited bleeding (*n* = 1) at the site of LAMS placement. There were five late adverse events that occurred in the dDEN group, including stent occlusion (*n* = 3), stent migration outside the cyst cavity (*n* = 1), and bleeding (*n* = 1) that occurred nine days post‐procedure secondary to pseudoaneurysm, requiring angioembolization.

A total of seven deaths in the iDEN group and four deaths in the dDEN group were seen at the 6‐month follow‐up (*p* = 0.53). Of the deaths in the iDEN group, four were attributed to clinical failure and three were due to unrelated causes. Of the four deaths in the dDEN group, one was due to clinical failure, and the remaining three were attributed to unrelated causes (Table 5).

**TABLE 5:** Summary of patients’ mortality at 6‐month follow‐up after endoscopic drainage of walled‐off necrosis

Mortality at 6 months (*n* = 11)	
iDEN (*n* = 7)	
Clinical failure	4
Unrelated causes	3
End‐stage COPD	1
Advanced heart failure	2

dDEN (*n* = 4)	
Clinical failure	1
Unrelated causes	3
Pulmonary hemosiderosis	1
Hyperosmolar hyperglycemic syndrome	1
Advanced dementia	1

Abbreviations: COPD, chronic obstructive pulmonary disease; dDEN, delayed direct endoscopic necrosectomy; iDEN, immediate direct endoscopic necrosectomy.

There was a total of 3 recurrences in the dDEN group at the 6‐month follow‐up. One patient underwent successful endoscopic reintervention. The other two patients were asymptomatic and were managed conservatively with close follow‐up and serial imaging. No recurrences were seen in the iDEN group at 6 months.

## DISCUSSION

Endoscopic cystgastrostomy was historically performed with the placement of double pigtail plastic stents (DPS). These stents were inexpensive and safe to use, however they were limited by their small stent diameter and high risk of occlusion.^13^, ^14^ In addition, DEN following DPS placement was cumbersome and required frequent tract dilations to advance the scope into the cyst cavity. The advent of LAMS has provided advantages over plastic stents including its large stent diameter, single‐step deployment, easy access to the cyst cavity, and decreased frequency of stent occlusion.^15^ This has placed endoscopic transmural drainage with LAMS at the forefront of WON management.

Direct endoscopic necrosectomy has improved clinical outcomes for the management of WON over traditional percutaneous and surgical drainage. While these techniques have been evaluated for their efficacy, the ideal endoscopic approach including the timing of DEN has yet to be established. Many endoscopists perform DEN during the same procedure as LAMS deployment.^16^ In contrast, some endoscopists perform necrosectomy on follow‐up endoscopy at least 1 week following initial stent placement. In a multicenter retrospective analysis by Yan et al., 271 patients treated with endoscopic necrosectomy were recruited from eight medical centers. The authors demonstrated that DEN performed at initial endoscopy resulted in a lower number of necrosectomy sessions for WON resolution, while procedure‐related adverse events, clinical success, and percentage of patients requiring surgical step‐up or percutaneous drainage were similar in the two groups (immediate vs. delayed DEN). The major limitation of this study was the variability in the technique of the endoscopists from different institutions along with differences in follow‐up intervals and cross‐sectional imaging quality.^12^

Our study did not corroborate a fewer number of endoscopic necrosectomies with iDEN. In fact, we showed that dDEN not only results in fewer interventions but also shorter index hospital length of stay in collections with greater than 10% necrosis. A recent randomized controlled trial by the Dutch pancreatitis group found that delayed catheter drainage for infected necrotizing pancreatitis resulted in fewer invasive interventions.^17^ While this study did not specifically look at DEN, the rationale for delaying intervention is likely similar. The large diameter of a LAMS allows gastric juices to enter the cyst cavity, aiding in the digestion of necrotic material and passive drainage. This in conjunction with IV antibiotics and hydrogen peroxide lavage avoids the inflammatory response associated with DEN and may play a crucial role in reducing the patients’ index hospital length of stay and the total number of necrosectomies.^6^, ^18^

Our study has notable strengths which may contribute to the differing results from the study by Yan et al. All procedures were performed in conjunction with a multidisciplinary team including interventional endoscopists, a hepatobiliary surgeon, and interventional abdominal radiologists at a large tertiary care center. Important variables were controlled for in the study design. There was an established procedural protocol that was followed by the endoscopists. This included a standardized diameter of LAMS, avoidance of DPS or nasocystic drains through the LAMS, identical debridement technique and tools, and uniform quantity and dilution of hydrogen peroxide used as adjunctive therapy in these patients. Furthermore, all follow‐up visits and imaging occurred at one institution.

The primary limitation of this study is the lack of patient randomization. The choice of iDEN or dDEN was based on the absence or presence of SIRS which may contribute to selection bias. Other limitations include the retrospective nature of the study and data from a single large tertiary care center making generalizability challenging.

In conclusion, we demonstrate the effective use of LAMS for endoscopic management of symptomatic walled‐off pancreatic necrosis with a preference toward dDEN. Further studies are needed to confirm these findings with a randomized controlled trial.

## CONFLICT OF INTEREST

None.

## FUNDING INFORMATION

None.
